# Underdetermined DOA Estimation Using MVDR-Weighted LASSO

**DOI:** 10.3390/s16091549

**Published:** 2016-09-21

**Authors:** Amgad A. Salama, M. Omair Ahmad, M. N. S. Swamy

**Affiliations:** Department of Electrical and Computer Engineering, Concordia University, Montreal, PQ H3G 1M8, Canada; am_adeli@encs.concordia.ca (A.A.S.); swamy@ece.concordia.ca (M.N.S.S.)

**Keywords:** adaptable LASSO, sparse array, direction of arrival estimation, compressive sensing, sensor array processing

## Abstract

The direction of arrival (DOA) estimation problem is formulated in a compressive sensing (CS) framework, and an extended array aperture is presented to increase the number of degrees of freedom of the array. The ordinary least square adaptable least absolute shrinkage and selection operator (OLS A-LASSO) is applied for the first time for DOA estimation. Furthermore, a new LASSO algorithm, the minimum variance distortionless response (MVDR) A-LASSO, which solves the DOA problem in the CS framework, is presented. The proposed algorithm does not depend on the singular value decomposition nor on the orthogonality of the signal and the noise subspaces. Hence, the DOA estimation can be done without a priori knowledge of the number of sources. The proposed algorithm can estimate up to $(\left(M^{2}-2\right)/2+M-1)/2$ sources using *M* sensors without any constraints or assumptions about the nature of the signal sources. Furthermore, the proposed algorithm exhibits performance that is superior compared to that of the classical DOA estimation methods, especially for low signal to noise ratios (SNR), spatially-closed sources and coherent scenarios.

## 1. Introduction

Array signal processing plays an important role in many applications, including sonar, radar, seismology and radio astronomy. Direction of arrival (DOA) deals with the problem of determining the number and locations of multiple sources using an antenna array. Many of the recent algorithms deal with DOA estimation of spatiotemporal electromagnetic waves emanating from multiple sources. Beamforming can be considered as a very early attempt at DOA estimation. This is based on mechanically steering an array to scan all possible angles in a sector of interest followed by measuring the output power of the array. The power spectrum will have a peak when the angle given by the mechanical steering corresponds to the DOA of one of the incoming source signals [1,2]. However, classical beamforming techniques fail to identify closely-separated source signals. Capon’s beamformer can overcome this problem; it maximizes the signal to noise ratio by using all of the available degrees of freedom to form a beam towards the interested signal source and at the same time makes null all of the other directions [3]. The linear prediction method minimizes the sensor array mean output power assuming that the weight on a selected sensor is unity [4]. Use of the maximum likelihood (ML) technique has been investigated for DOA estimation in [5]. ML leads to highly accurate DOA estimation results; however, ML methods are the most time consuming and complex DOA estimation algorithms among all of the DOA estimation techniques.

MUSIC [6] and ESPRIT [7] are two of the most studied algorithms in DOA estimation. The MUSIC algorithm is based on the eigen decomposition of the covariance matrix of the received data. Unlike conventional DOA techniques, it is easy to implement MUSIC with high resolution. However, it requires the number of sources to be known in advance. If the exact number of sources is unknown, this will lead to a failure of the DOA estimation.

ESPRIT essentially investigates the shift-invariance properties of the sensor array and is based on the assumption that the array is composed of two identical subarrays. The DOA can be estimated by studying the shift-invariance properties of the output of each sub-array. Unfortunately, like the MUSIC algorithm, ESPRIT also uses the eigenvalue decomposition and, hence, requires the number of sources to be known in advance [8,9].

Increasing the number of sensor array elements leads to an enhancement in the array gain and directivity, thus improving the array’s overall performance. On the other hand, increasing the number of array elements increases the complexity of the feeding network and the time needed to process the received data. Researchers have focused recently on the pursuit of light weight, small sized and compact antenna arrays to satisfy the requirements of present day applications like the Internet of Things (IoT) and unmanned vehicles [10]. Physically increasing the number of antenna array elements is therefore not an option. Recent literature investigating the virtual array concept has shown it to enhance the performance of a sensor array [11,12,13,14,15].

Minimum redundancy arrays (MRAs) and augmented covariance matrices (ACMs) have been investigated for virtually extending the sensor array aperture by increasing the available degrees of freedom [16,17,18]. However, there is no closed form expression for the locations of the sensors in the virtual array and for the number of achievable degrees of freedom. Furthermore, ACM is not positive semi-definite for a finite number of snapshots, and a transformation was suggested in [19] to overcome this problem. Higher order cumulants were used to increase the available degrees of freedom and consequently extend the array aperture. However, using the fourth order cumulants, Gaussian source signals cannot be estimated. The Khatri-Rao (KR) product was suggested for the DOA estimation of quasi-stationary source signals in [12,13].

Co-prime arrays have been proposed in the literature [20,21,22,23] to increase the degrees of freedom in the array. For these arrays, the number of the degrees of freedom can be determined, and closed-form expressions for the locations of the sensors in the corresponding virtual arrays can be obtained. However, the corresponding virtual arrays are not uniform. Nested arrays have been investigated for the first time in [14]. These arrays, in addition to all of the advantages provided by the co-prime arrays, result in virtual arrays that are uniform and provide degrees of freedom that are even higher [23]. For example, using six elements, the nested array provides 23 degrees of freedom in contrast to only 17 provided by the co-prime array.

Compressive sensing (CS) has evolved rapidly in recent years and has found multiple applications in various fields, such as medicine [24], ultrasound imaging [25] and radar detection [26]. Malioutov et al. [27] investigated the DOA estimation performance using CS with respect to the signal to noise ratio, the number of sources and the coherence of the sources’ signals. Xenaki et al. [28] studied the DOA estimation using CS with coherent arrivals, single-snapshot data and different array geometries. It has been proven in [28] that CS does not require the arrivals to be incoherent. Furthermore, single or multiple snapshots can be used. In terms of resolution, the performance of the CS-based DOA is superior to that of the minimum variance distortionless response (MVDR) [29].

The least square (LS) method is based on minimizing the residual squared error. LS estimates have low bias and large variance; therefore, LS estimates suffer from low prediction accuracy, which can be enhanced through setting some coefficients to zero. Furthermore, LS estimates can be improved by subset selection and ridge regression; however, both of these have some drawbacks. Subset selection can provide models that are interpretable. However, small changes in the data can lead to very different models being selected. Ridge regression is more stable, but is not an interpretable model [30].

The least absolute shrinkage and selection operator (LASSO) [30] minimizes the residual sum of squares (subject to the sum of the absolute values of the coefficients being less than a constant), and it is popular for solving CS problems. The LASSO technique combines the advantages of both subset selection and ridge regression. Zou [31] proposed a new version of LASSO, whereby adaptable weights are used for penalizing different coefficients in the $\ell_{1}$ penalty function. Panahi et al. [32] discussed the resolution of the LASSO-based DOA estimation; it has been noted that the LASSO-based DOA estimation is better than that of the traditional beamforming techniques. It should be pointed out that the above-mentioned techniques use uniform arrays, and some of them require the number of signal sources to be known in advance, especially when singular value decomposition (SVD) of the covariance matrix of the received data is performed [27].

In this paper, we propose a new algorithm for DOA estimation, the MVDR A-LASSO, which combines the benefits of the virtual array concept for extended array aperture [12,14] along with CS. In Section 2, we introduce the co-array principle and apply it to a sparse array. The CS framework is presented in Section 3. In Section 4, a modified version of the adaptable LASSO, MVDR A-LASSO, is proposed in order to further improve the performance. In Section 5, the performance of the proposed MVDR A-LASSO is studied using simulations, and finally, conclusions are drawn in Section 6.

### Notations

Superscript ${}^{H}$ denotes conjugate transpose; superscript ${}^{*}$ denotes conjugation without transpose; and ${}^{T}$ denotes the transpose operation. The symbol ⊙ denotes the Khatri-Rao (KR) product [33] between two matrices of appropriate sizes.

## 2. Difference Co-Array

Consider a linear array (LA), uniform or non-uniform, consisting of *M* elements. Let $d_{i}$ denote the *i*-th element position in the array. Let us assume that there are *L* narrowband, far-field sources with angles-of-arrival (AOA) $\left(\theta_{l}\right)$ and powers $\left(\sigma_{l}^{2}\right)$, l=1,2,…,L. It is also assumed that the source signals are uncorrelated with one another. Let $a\left(\theta_{l}\right)\in C^{M\times 1}$ be the steering vector corresponding to AOA $\left(\theta_{l}\right)$, whose *i*-th element is $e^{-jk_{o}d_{i}\cos\left(\theta_{l}\right)}$, where $k_{o}=2\pi /\lambda$ is the wavenumber and *λ* is the wavelength of the propagating waves. Let the vector $s\left(t\right)={\left[s_{1}\left(t\right)\;s_{2}\left(t\right)\;\ldots \;s_{L}\left(t\right)\right]}^{T}$, where $s\in C^{L\times 1}$ represent the source signals. Then, the output of LA can be written as:

(1) x(t)=As(t)+n(t)

where $A=[a\left(\theta_{1}\right)\;a\left(\theta_{2}\right)\;\ldots \;a\left(\theta_{L}\right)]$, $A\in C^{M\times L}$ is the array manifold matrix and $n\left(t\right)\in C^{M\times 1}$ is an additive white Gaussian noise (AWGN) that is uncorrelated with the source signals. One can obtain the covariance matrix of the received signals as [34]:

(2) $$\begin{array}{cc} R_{xx} & =E[{\mathrm{xx}}^{H}]\\ & =AR_{ss}A^{H}+\sigma_{n}^{2}I\\ & =A\left[\begin{array}{ccccc} \sigma_{1}^{2}\\ & \ddots \\ & & \sigma_{l}^{2}\\ & & & \ddots \\ & & & & \sigma_{L}^{2} \end{array}\right]A^{H}+\sigma_{n}^{2}I \end{array}$$

where $\sigma_{l}^{2},\;l=1,\ldots ,L$, correspond to the power of the source signals, I is the identity matrix of size (M×M) and $\sigma_{n}^{2}$ is the noise power. One can now vectorize $R_{xx}\in C^{M\times M}$ as [12,13]:

(3) $$\begin{array}{cc} V & =\mathrm{vec}\left(R_{xx}\right)=\mathrm{vec}\left[\sum_{l=1}^{L}\sigma_{l}^{2}\left(a\left(\theta_{l}\right)a^{H}\left(\theta_{l}\right)\right)\right]+\sigma_{n}^{2}1\\ & =\left(A^{*}⊙A\right)p+\sigma_{n}^{2}1 \end{array}$$

where $p\in C^{L\times 1}={\left[\sigma_{1}^{2}\;\sigma_{2}^{2}\;\ldots \;\sigma_{L}^{2}\right]}^{T}$ and $1\in C^{M\times M}={\left[e_{1}^{T}\;e_{2}^{T}\;\ldots \;e_{M}^{T}\right]}^{T}$ with $e_{i}\in C^{M\times 1}$ being a column vector of zeros except for a one at the *i*-th position. Comparing Equations (3) and (1), we can see that $V\in C^{M^{2}\times 1}$ in Equation (3) can be considered as the output of an array with a manifold $\left(A^{*}⊙A\right)$, p representing the equivalent source signals and the noise given by $\sigma_{n}^{2}1$ being deterministic. The distinct rows of $\left(A^{*}⊙A\right)$ form the virtual array (VA); the locations of whose distinct elements are given by the set:

(4) $$D=\left\{d_{i}-d_{j}\right\},\;\forall i,j=1,2,\ldots ,M$$

where $d_{i}$ is the position vector of the *i*-th sensor in the original array. This array is known as the difference co-array [14].

We now assume that the original array is a two-level nested array [14] for which *M* is even, and each level contains M/2 sensor elements. In such a case, the VA is a uniform linear array (ULA) consisting of $\bar{M}$ sensors, $\bar{M}=\left(M^{2}/2+M-1\right)$, which are located from $-(\bar{M}-1)d/2$ to $(\bar{M}-1)d/2$, where *d* is the distance between two adjacent sensors [14]. It should be noted that the equivalent source signal vector p (for the difference co-array) contains the power of the sources $\sigma_{l}^{2}$, l=1,…,L. Therefore, they act like fully-correlated sources. A spatial smoothing technique was suggested by Pal et al. [14] to overcome this problem of correlated sources. However, by using the CS technique, we will no longer need spatial smoothing or any preprocessing scheme, and CS will be able to detect the source signals.

## 3. Compressive Sensing Framework

Since the sources are assumed to be located in the far field, they can be considered as point sources; hence, the sources become sparse in space. Let Ω denote the set of all possible source locations, ${\left\{{\bar{\theta }}_{n}\right\}}_{n=1}^{N}$, *N* denoting a grid that covers Ω, with N≫L. Let:

(5) $$\bar{s}\left(t\right)={\left[{\bar{\sigma }}_{1}\;{\bar{\sigma }}_{2}\;\ldots \;{\bar{\sigma }}_{n}\;\ldots \;{\bar{\sigma }}_{N}\right]}^{T}$$

where $\bar{s}\in C^{N\times 1}$, and:

(6) $$\begin{array}{cc} \Phi & =[\bar{a}\left({\bar{\theta }}_{1}\right)\;\bar{a}\left({\bar{\theta }}_{2}\right)\;\ldots \;\bar{a}\left({\bar{\theta }}_{n}\right)\;\ldots \;\bar{a}\left({\bar{\theta }}_{N}\right)]\\ & =\left[\begin{array}{cccc} e^{jk_{o}d\left(-\left(\bar{M}-1\right)/2\right)\cos{\bar{\theta }}_{1}} & e^{jk_{o}d\left(-\left(\bar{M}-1\right)/2\right)\cos{\bar{\theta }}_{2}} & \cdots & e^{jk_{o}d\left(-\left(\bar{M}-1\right)/2\right)\cos{\bar{\theta }}_{N}}\\ \vdots & \vdots & & \vdots \\ 1 & 1 & \ddots & 1\\ \vdots & \vdots & & \vdots \\ e^{jk_{o}d\left(\left(\bar{M}-1\right)/2\right)\cos{\bar{\theta }}_{1}} & e^{jk_{o}d\left(\left(\bar{M}-1\right)/2\right)\cos{\bar{\theta }}_{2}} & \cdots & e^{jk_{o}d\left(\left(\bar{M}-1\right)/2\right)\cos{\bar{\theta }}_{N}} \end{array}\right] \end{array}$$

where $\Phi \in C^{\bar{M}\times N}$ and $\bar{a}\left({\bar{\theta }}_{n}\right)\in C^{\bar{M}\times 1}$ is the steering vector of the VA corresponding to the AOA $\left({\bar{\theta }}_{n}\right)$. Then, the received signal at the $\bar{m}$-th sensor is:

(7) $$y_{\bar{m}}\left(t\right)=\phi_{\bar{m}}\bar{s}\left(t\right)+{\bar{n}}_{\bar{m}}\left(t\right),\;\bar{m}=1,2,\ldots ,\bar{M}$$

where $\phi_{\bar{m}}$ is the $\bar{m}$-th row of Φ. The *n*-th element of $\bar{s}\left(t\right)$, ${\bar{s}}_{n}\left(t\right)$, is nonzero only if (${\bar{\theta }}_{n}=\theta_{l}$), and in that case, ${\bar{\sigma }}_{n}=\sigma_{l}$. Then, Equation (7) can be rewritten as:

(8) $$y\left(t\right)=\Phi \bar{s}\left(t\right)+\bar{n}\left(t\right)$$

where $y\in C^{\bar{M}\times 1}$ and $\bar{n}\in C^{\bar{M}\times 1}$. In accordance with conventional DOA estimation, the technique is to estimate the signal energy as a function of the source location showing peaks corresponding to the source locations. Since the sources are point sources and their number is small, the spatial spectrum is sparse. Hence, we can solve this problem by regularizing it to favour sparse signal fields using LASSO [30]. The LASSO minimization is defined as:

(9) $$\min_{s}{∥y-\sum_{n=1}^{N}\phi_{n}{\bar{s}}_{n}∥}^{2}+\tau \sum_{n=1}^{N}\left|{\bar{s}}_{n}\right|$$

where $\phi_{n}$ is the *n*-th element of $\phi_{\bar{m}}$ and ${\bar{s}}_{n}$ is the *n*-th element of $\bar{s}$. Equation (9) can be rewritten as:

(10) $${\hat{s}}_{lasso}=\min_{s}{∥y-\Phi \bar{s}∥}_{2}^{2}+\tau {∥\bar{s}∥}_{1}$$

where *τ* is a nonnegative regularization parameter. The first term in Equation (9) is the $\ell_{2}$ norm, while the second is an $\ell_{1}$ penalty function, which is very important for the success of LASSO. LASSO shrinks the coefficients toward zero, as the regularization parameter *τ* increases. This parameter, *τ*, controls the relative importance between the sparsity of the solution ($\ell_{1}$-norm term) and the fitness to the measurements ($\ell_{2}$-norm term). However, the $\ell_{1}$-norm penalty associated with LASSO tends to produce biased estimates for large coefficients [35], thus degrading the estimation accuracy. Zou [31] proposed a new version of LASSO, the adaptable LASSO (A-LASSO), wherein adaptable weights are used for penalizing the coefficients in the $\ell_{1}$-norm term iteratively. Furthermore, Zou [31] suggests using the ordinary least squares (OLS) solution as the initial weights to construct the adaptable weights in the adaptable LASSO first iteration. We shall refer to this as OLS A-LASSO. It should be mentioned that the $\ell_{1}$ penalization approach is also known as basis pursuit [36].

## 4. Modified LASSO for DOA Estimation

One can notice from Equation (9) that the regularization parameter, *τ*, penalizes the coefficients equally in the $\ell_{1}$-norm term. Therefore, the LASSO estimates could be biased [35] and result in reducing the solution accuracy. In order to overcome this deficiency, we apply the A-LASSO in the DOA estimation problem for the first time. Hence, the A-LASSO minimizes:

(11) $${∥y-\Phi \bar{s}∥}_{2}^{2}+\tau \sum_{n=1}^{N}w_{n}\left|{\bar{s}}_{n}\right|$$

where $w_{n}$ is the *n*-th element of the weight vector, $w\in C^{N\times 1}$. Let, $\hat{s}$ be the initial estimate for $\bar{s}$. Now, choosing any weight factor, *γ*, where γ>0, and defining the weight vector as $\hat{w}={\left[{\hat{w}}_{1}{\hat{w}}_{2}\ldots {\hat{w}}_{N}\right]}^{T}$, where:

(12) $${\hat{w}}_{n}=\frac{1}{|{\hat{s}}_{n}{|}^{\gamma }}\;n=1,2,\ldots ,N$$

the A-LASSO is given by:

(13) $${\hat{s}}^{\left(k\right)}=\min_{s}{∥y-\Phi \bar{s}∥}_{2}^{2}+\tau_{k}\sum_{n=1}^{N}{\hat{w}}_{n}\left|{\bar{s}}_{n}\right|$$

where *k* is the iteration number and ${\hat{w}}_{n}$ is the *n*-th element of the weight vector, $\hat{w}$. The minimization in Equation (13) corresponds to a convex optimization problem; it does not have multiple local minima, and its global minimizer can easily be found. The A-LASSO is $\ell_{1}$ penalized, so any efficient algorithm that can solve the conventional LASSO should also be able to solve the adaptable version. The least angle regression (LARS) algorithm [37] is utilized to solve the A-LASSO using the following steps:

1. Let the initial estimate for $\bar{s}$ be $\hat{s}$.
2. Find $\hat{w}$, where the *n*-th element of $\hat{w}$, ${\hat{w}}_{n}$, is given by ${\hat{w}}_{n}=\frac{1}{|{\hat{s}}_{n}{|}^{\gamma }},\;n=1,2,\ldots ,N$.
3. Define $\bar{M}\times N$ matrix $\Phi^{*}$, such that its $\left(\bar{m},n\right)$-th element is given by $\phi_{\bar{m}n}/{\hat{w}}_{n}$, where $\bar{m}=1,2,\ldots ,\bar{M}$ and n=1,2,…,N.
4. Solve the LASSO problem as:
  $${\hat{s}}^{*}=\min_{s}{∥y-\Phi^{*}\bar{s}∥}_{2}^{2}+\tau_{k}{∥\bar{s}∥}_{1}$$
5. Calculate ${\hat{s}}^{\left(k\right)}={\hat{s}}_{n}^{*}/{\hat{w}}_{n},\;n=1,2,\ldots ,N$.

**Figure 1:**
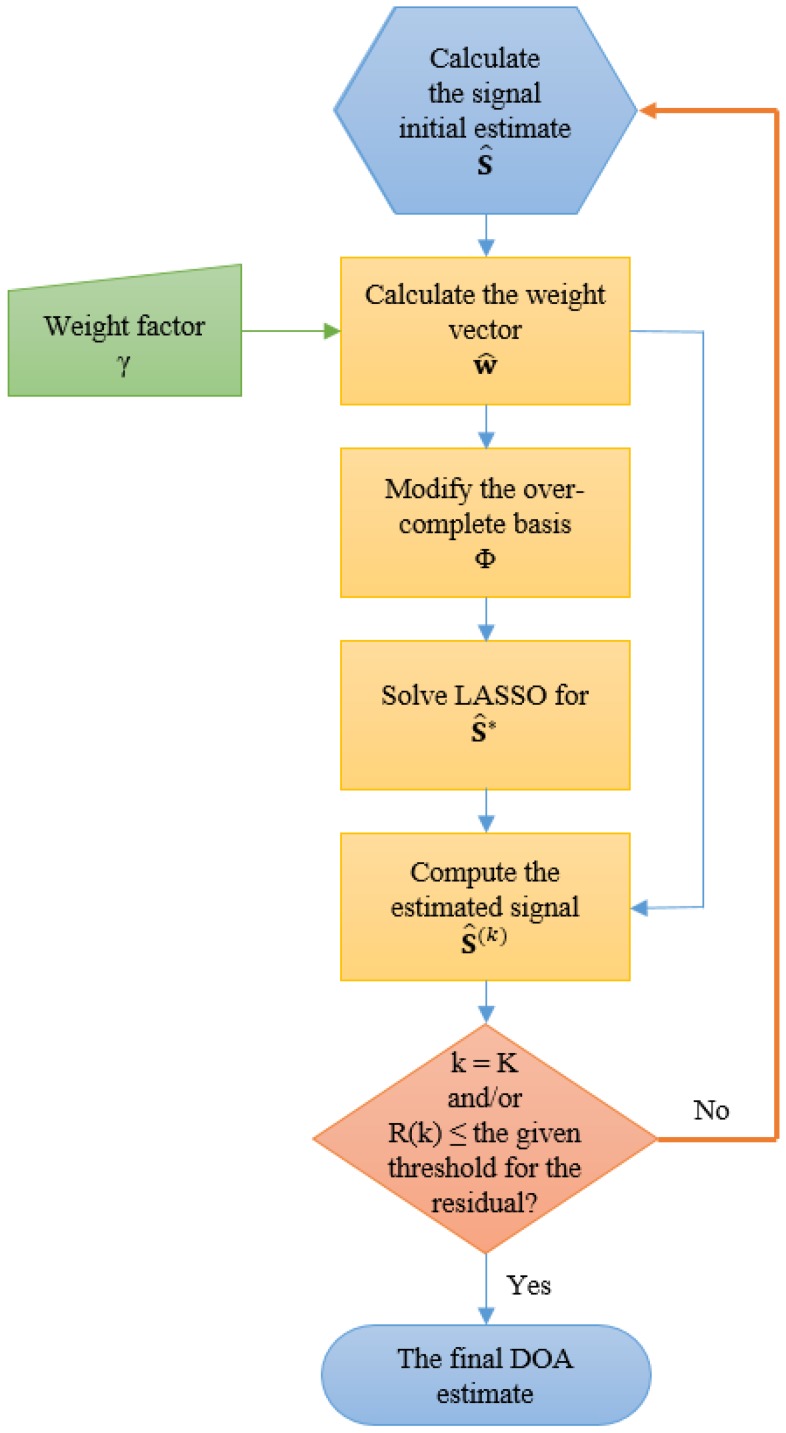
A flowchart of the algorithm for adaptable (A)-LASSO-based DOA estimation.

The steps from 2 to 5 are repeated until convergence to a predefined residual, *R*, is obtained or when the chosen number of iterations is reached. The computational cost is of the order $O(KN^{2})$, where *K* is the total number of iterations, which is of the same order as the computation of a single OLS minimization. Figure 1 summaries the above steps from 1 to 5. The efficient path algorithm makes the A-LASSO an attractive method for practical applications [31].

For the uniqueness of the sparse solution, the spark of matrix Φ, defined as the smallest number of columns from Φ that are linearly dependent [38], must be investigated. Hence, the above algorithm can identify a unique *L*-sparse solution only if L<Spark[Φ]/2, Spark[.] denoting the spark of a matrix [38]. Since any set of $\left(M^{2}/2+M-1\right)$ columns of Φ is linearly independent, $Spark\left(\Phi \right)=\left(M^{2}/2+M\right)$. Hence, the algorithm can identify *L*-sparse solutions only if L<Spark(Φ)/2. That is, our algorithm can detect up to $(M^{2}/2+M-2)/2$ sources using an array of *M* sensors.

### 4.1. OLS A-LASSO

Previously, a vector of ones is assumed as the initial signal estimate for $\bar{s}$. However, as proven from the simulations, a vector of ones is not the appropriate guess for the signal to be estimated, especially since there is no relation between the vector of ones and the signal to be estimated. Furthermore, multiplying that vector by a factor, *β*, will affect the regularized solution (the same effect as that of changing *τ*, the regularization parameter). Even more, if we try to push the algorithm to the limits (by choosing a small *β*), we find spurious peaks along with the genuine peaks. Therefore, we will use the OLS solution as the initial signal estimate for $\bar{s}$, with the expectation that this modification leads to better results and that the OLS A-LASSO solution converges faster than that of the A-LASSO.

We assume replacing ${\hat{s}}_{n}$ in Equation (12) with $\hat{s}{{}_{OLS}}_{n}$, where $\hat{s}{{}_{OLS}}_{n}$ is the *n*-th element of ${\hat{s}}_{OLS}\in C^{N\times 1}$ and is given by:

(14) $${\hat{s}}_{OLS}=\min_{s}{∥y-\Phi \bar{s}∥}_{2}^{2}$$

The minimization in Equation (14) is known as the ordinary least square minimization. The computational cost for Equation (14) is of order $O(N^{2})$. It should be mentioned that the number of source signals is not required to be known in advance for OLS A-LASSO. However, we do not use OLS for DOA estimation. We use it in OLS A-LASSO only as an initial guess for the signal to be estimated (not a stand-alone DOA estimation technique). Furthermore, OLS gives nonzero estimates to all of the coefficients (compared to LASSO minimization) and does not favour sparse signals as in the case of LASSO minimization.

### 4.2. MVDR A-LASSO

The MVDR technique uses the available degrees of freedom to form a beam in the look direction and, at the same time, nulling the output in all of the other directions. Thus, for a particular DOA, MVDR uses all, but one of the degrees of freedom to minimize the array output while using the remaining ones to constrain a unity gain in the look direction according to the following optimization [2]:

(15) $$\min_{z}\;z^{H}R_{ss}z,\;\text{subject to}\;z^{H}a_{1}\left(\bar{\theta }\right)=1$$

where $z\in C^{(M^{2}/4+M/2)\times 1}$ is the MVDR beamformer weight vector, $R_{ss}$ is the spatially-smoothed (SS) covariance matrix, which we will now obtain, and $a_{1}\in C^{(M^{2}/4+M/2)\times 1}$ is the steering vector of the array whose SS covariance matrix is $R_{ss}$. It should be noted that we are not able to use the covariance matrix $R_{xx}$ in Equation (2), since this contains information only about the real sensor array. Furthermore, the received source signals are represented as the deterministic vector **p** in Equation (3). Therefore, we are not able to use **V** of Equation (3) directly for MVDR, since the resultant covariance matrix is rank defective. However, in our case, it is required to construct the covariance matrix of the virtual array. Hence, we perform spatial smoothing on **V** to construct a full rank covariance matrix for the virtual array. Assuming a two-level nested array containing *M* even sensors, the distinct elements of vector V in Equation (3), $\bar{V}\in C^{\bar{M}\times 1}$ can be rewritten as:

(16) $$\bar{V}={\left[{\bar{v}}_{1}\;{\bar{v}}_{2}\;\ldots \;{\bar{v}}_{\bar{m}}\;\ldots \;{\bar{v}}_{\bar{M}}\right]}^{T},\;\bar{M}=\left(M^{2}/2+M-1\right)$$

Then, the covariance matrix of the virtual array can be obtained as follows. Let R be the Toeplitz matrix:

(17) $$R=\left[\begin{array}{cccc} {\bar{v}}_{\frac{\left(\bar{M}-1\right)}{2}+1} & {\bar{v}}_{\frac{\left(\bar{M}-1\right)}{2}+2} & \ldots & {\bar{v}}_{\bar{M}}\\ {\bar{v}}_{\frac{\left(\bar{M}-1\right)}{2}} & {\bar{v}}_{\frac{\left(\bar{M}-1\right)}{2}+1} & \ldots & {\bar{v}}_{\bar{M}-1}\\ \vdots & \vdots & \ddots & \vdots \\ {\bar{v}}_{1} & {\bar{v}}_{2} & \ldots & {\bar{v}}_{\frac{\left(\bar{M}-1\right)}{2}+1} \end{array}\right]$$

where $R\in C^{M^{2}/4+M/2\times M^{2}/4+M/2}$. Forward-backward (FB) SS [34] is applied to R to obtain the spatial smoothed covariance matrix, $R_{ss}$. It should be noted that FBSS is used here only in establishing a full rank covariance matrix. The resulting *n*-th element of the weight vector $w_{MVDR}\in C^{N\times 1}$ is given by [2]:

(18) $$w\left({\bar{\theta }}_{n}\right)=\frac{1}{a_{1}^{H}\left({\bar{\theta }}_{n}\right)R_{ss}^{-1}a_{1}\left({\bar{\theta }}_{n}\right)},\;n=1,2,\ldots ,N$$

which is also known as the scalar output power for a single steering direction [39]. The computational complexity of the MVDR algorithm [40] is as shown in Table 1. It is noted that the MVDR-based DOA estimation technique does not require the number of the source signals to be known in advance. Furthermore, the MVDR DOA estimation method performance is better than that of the conventional beamforming. In addition, assuming that ($N\gg \bar{M}$), the computational complexity of obtaining the MVDR weights is less than that of obtaining the OLS one.

It should be noted that our proposed algorithm does not depend on the orthogonality of the signal subspaces nor on implementing singular value decomposition (SVD) on the sensor array data. Therefore, it can perform DOA estimation without knowing the number of source signals in advance. On the other hand, subspace-based techniques, such as MUSIC and ESPRIT, cannot estimate the DOA without a priori knowledge of the number of source signals. Furthermore, it is known from the literature that the MUSIC algorithm is superior to the ESPRIT algorithm [41,42,43]. However, for the sake of evaluating our proposed algorithm in comparison with MUSIC, we assume that the number of signal source is to be known a priori.

### 4.3. Wideband MVDR A-LASSO DOA

Recently, wideband wireless applications have dominated the area of wireless communications. Orthogonal frequency division multiplexing (OFDM) transmission and ultra-wide band (UWB) systems are the most-used techniques in that field. DOA of wideband signals is required in such applications. However, there are only a few techniques that are applicable to the wideband scenario [44,45]. When DOA of wideband source signals needs to be estimated, we cannot represent the delays of the signals by simple phase shifts. An easy way to overcome this problem is to decompose the wideband signals into several narrowband ones and apply the DOA scheme for each of the narrowband signals. A filter-bank (or Fourier transform) consisting of *Q* filters could be used to decompose the wideband signal into the narrowband ones, where each filter $h_{q}\left(t\right)$ has a narrowband around the central frequency $f_{q},\;q=1,2,\ldots ,Q$. Thus, our DOA estimation is essentially equivalent to estimating the DOA of *Q* narrowband signals, given by:

(19) $$y_{q}\left(t\right)=\Phi \left(f_{q}\right){\bar{s}}_{q}\left(t\right)+n_{q}\left(t\right)\;q=1,2,\ldots ,Q$$

The over-complete steering matrix, $\Phi (f_{q})$, is computed for each filter centre-frequency, $f_{q}$.

(20) $$f_{q}=\frac{{\overset{ˇ}{f}}_{q}+{\hat{f}}_{q}}{2}$$

where ${\overset{ˇ}{f}}_{q}$ and ${\hat{f}}_{q}$ are the lower and higher cut-off frequencies of the filters. It should be noted that in [45], the KR product is used to virtually extend the array aperture, thus resulting in an increase of the available degrees of freedom. Consequently, the algorithm proposed in [45] can detect more source signals than the number of sensors used. However, our proposed technique can detect more source signals than that of [45]. Moreover, the technique proposed in [45] cannot detect correlated source signals (unless they are decorrelated before DOA estimation); but, our proposed technique can identify correlated source signals. In addition, as will be demonstrated in the last experiment of Section 6, our proposed technique can estimate the DOAs of the source signals even for a wideband scenario with a very much smaller number of snapshots compared to that required with the technique in [45].

### 4.4. Selecting the Regularization Parameter

**Figure 2:**
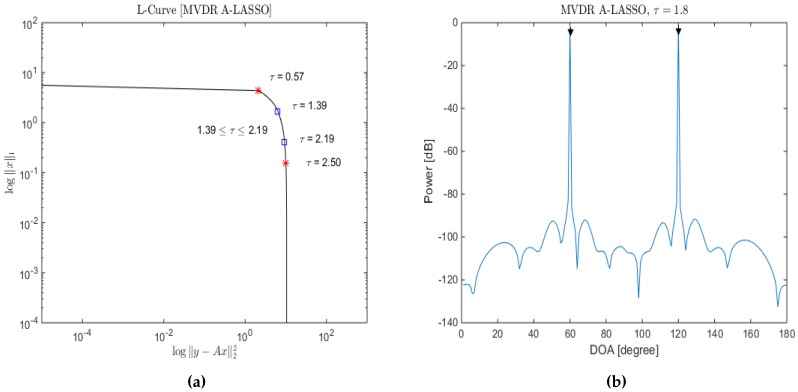
(**a**) The data residual ${∥y-\Phi^{*}\bar{s}∥}_{2}^{2}$ versus the solution $\ell_{1}$-norm linear scale on a log-log scale (L-curve); (**b**) DOA estimation for two source signals; *τ* was selected using L-curve, in the MVDR A-LASSO problem (SNR =10 dB).

**Figure 3:**
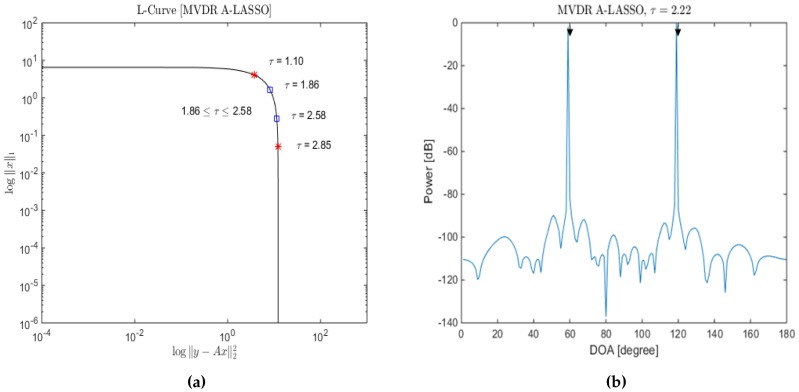
(**a**) The data residual ${∥y-\Phi^{*}\bar{s}∥}_{2}^{2}$ versus the solution $\ell_{1}$-norm linear scale on a log-log scale (L-curve); (**b**) DOA estimation for two source signals; *τ* was selected using L-curve, in the MVDR A-LASSO problem (SNR =0 dB).

Choosing the regularization parameter, *τ*, is an important issue for the success of LASSO minimization Equation (10). The regularization parameter controls the trade-off between the data fidelity (${∥y-\Phi \bar{s}∥}_{2}^{2}$) and the prior information (${∥\bar{s}∥}_{1}$). The discrepancy principle (DP), cross-validation (CV), generalized cross-validation (GCV) and L-curve method are some of the existing regularization parameter selection methods. The regularization parameter in DP is chosen so that the sum of squares of the weighted residuals is equal to the mean of a chi-square distribution [46,47,48]. CV selects the regularization parameter that minimizes the mean square error, while GCV selects the value of the regularization parameter that minimizes the GCV function, which is a leave-one-out CV function for large-scale problems [48,49]. The L-curve criterion is based on a log-log plot of the corresponding values of the solution norms and the residuals for a range of values of the regularization parameter [29,50,51]. From Figure 2a and Figure 3a, it is seen that as the value of the regularization parameter *τ* is increased, the significance of the A-LASSO estimates shifts from large non-sparse estimates to smaller sparse estimates. In other words, a small value of *τ* leads to an under-regularized estimate, whereas a large value results into an over-regularized estimate. Therefore, suitable values of *τ* are those lying in the knee of the L-curve. We consider the beginning of the knee to correspond the value of *τ* at which the solution norm starts to decrease and the end of the knee to correspond to the value of *τ* at which the residual norm does not significantly change. These are the two red stars on the L-curve. We empirically determine a segment of this knee corresponding to which all of the *τ* values provide satisfactory estimates. We have chosen the value of the regularization parameter *τ* to be the midpoint of this segment. It should be mentioned that there are different methods to select a suitable value for the regularization parameter in the literature [46,47,48,49]. However, it has been shown in [52] that the L-curve [29,50,51] method gives a good estimation of the regularization parameter. In order to illustrate how *τ* is chosen, we consider two source signals from DOAs of $60^{\circ }$and $120^{\circ }$ to be impinging a six-sensor two-level nested array with the sampling grid being uniform from $1^{\circ }$ to $180^{\circ }$, in increments of $1^{\circ }$, and an SNR of 10 dB; the corresponding L-curve plot is as shown in Figure 2a. Selecting *τ* to be between 1.39 and 2.19, the resulting DOA estimation is as shown in Figure 2b. Lowering the SNR to be 0 dB, the results for the same specifications are shown in Figure 3. In this case, a suitable value for *τ* is between 1.86 and 2.58. From Figure 2b, it can be seen that we are able to identify correctly the two source signals, even at low SNR conditions.

## 5. Simulation Results

**Figure 4:**
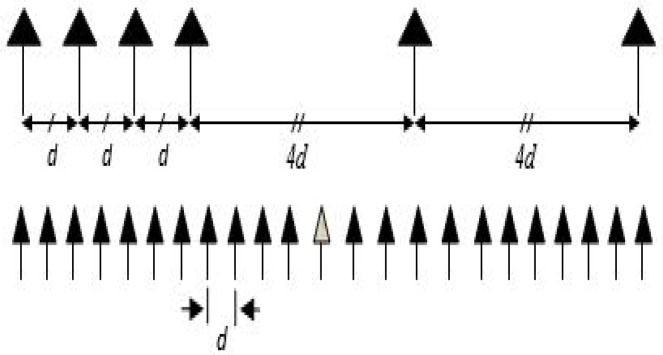
The proposed sparse (**upper**) and the virtual co-array (**lower**).

Consider a sparse linear two-level nested array, for which *M* is even, consisting of M=6 elements, as shown in Figure 4. Investigating the array output by applying Equations (1) to (3) and extracting the equivalent distinct virtual elements from the virtual array manifold $\left(A^{*}⊙A\right)$, one can see that the virtual uniform linear array (ULA) contains $\bar{M}=23$ elements, as shown in Figure 4. It should be noted that the resultant virtual is a ULA [14]. The sampling grid ${\bar{\theta }}_{n}\in \left[1^{\circ }:180^{\circ }\right]$ that covers Ω is chosen to be of $1^{\circ }$ step, except for the twelfth simulation and d=λ/2, where *λ* is the wavelength of the propagating waves.

All of the simulated source signals are assumed to be equi-power and uncorrelated with one another or with the noise, except in the fifth simulation, where the sources are assumed to be correlated. The weight parameter, *γ*, is set to 0.5 in all of the simulations, except for the eleventh simulation. The total number of simulations, $N_{sim}$, is set to $N_{sim}=100$ for each observation point except in the case of the eleventh simulation, where it is set to 10. For each simulation, the regularization parameter, *τ*, is selected based on the idea of the L-curve [29,50,51]. For the wideband scenario, two chirp signals are assumed for the wideband simulations, and the filter-bank is assumed to contain 10th order Butterworth bandpass filters.

The CVX toolbox [53,54] for convex optimization that is available within the MATLAB environment is used for examining the performance of the proposed A-LASSO algorithms. It uses semi-definite quadratic-linear programming (SDPT3) [55] to obtain the global solution for the optimization problem.

The root mean square error (RMSE) is used as the performance measure:

(21) $$RMSE=\frac{1}{L}\sum_{l=1}^{L}\sqrt{\frac{1}{N_{sim}}\sum_{n=1}^{N_{sim}}{\left({\hat{\theta }}_{l,n}-\theta_{l}\right)}^{2}}$$

where ${\hat{\theta }}_{l,n}$ is the estimate of the DOA angle $\theta_{l}$ of the *n*-th Monte Carlo trial.

### 5.1. Narrowband Signal Sources

#### 5.1.1. Investigations of LASSO-Based Algorithms

**Figure 5:**
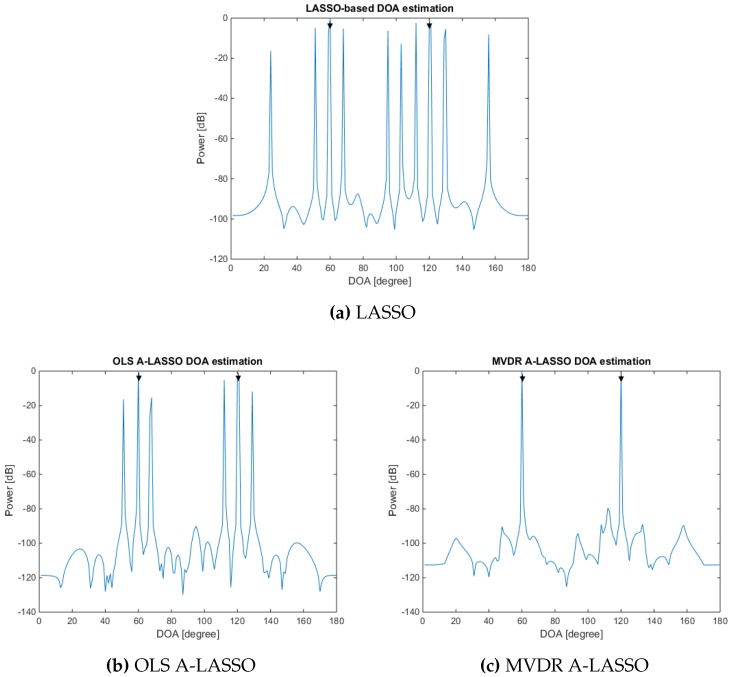
Performance of LASSO, OLS A-LASSO and MVDR A-LASSO, for two source signals at DOAs $60^{\circ }$ and $120^{\circ }$, 10 snapshots, SNR =0 dB and one iteration. (**a**) LASSO; (**b**) OLS A-LASSO; and (**c**) MVDR A-LASSO.

In the first simulation, we study the effect of the initial vectors on the performance of the three LASSO algorithms, namely classical LASSO (for which γ=0), OLS A-LASSO and MVDR A-LASSO. For the latter two, we assume γ=0.5. We consider two source signals impinging on the sparse array from the DOA of $60^{\circ }$ and $120^{\circ }$. For SNR of 0 dB, 10 snapshots and one iteration, the results are as shown in Figure 5. It can be seen that the MVDR A-LASSO yields a performance better than that of the classical LASSO, as well as that of the OLS A-LASSO. It may be mentioned that by increasing the number of iterations, OLS A-LASSO can be made to yield a performance similar to that of MVDR A-LASSO. This superior performance of the MVDR A-LASSO can be attributed to the initial weights used, compared to the least square weights used for the OLS A-LASSO, as will be seen later in Simulation 6. It should be noted that classical LASSO uses equal initial weights and has the poorest performance.

**Figure 6:**
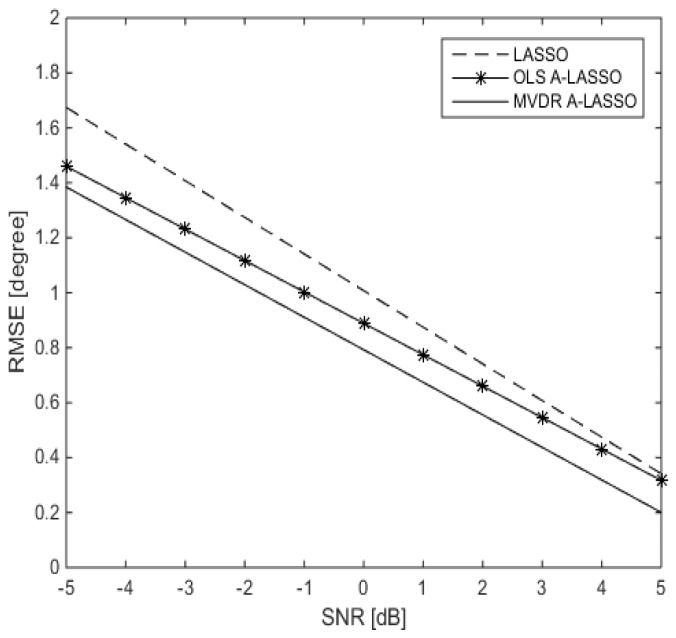
Performance of the three LASSO algorithms versus SNR, for two source signals at DOAs $60^{\circ }$ and $120^{\circ }$, 10 snapshots and after one iteration of the MVDR A-LASSO and OLS A-LASSO algorithms.

In the second simulation, we investigate the performance of the proposed MVDR A-LASSO algorithm as we vary SNR and compare it with that of LASSO and OLS A-LASSO. Two source signals are assumed to impinge on the sparse array from DOA of $60^{\circ }$ and $120^{\circ }$. The performances of the proposed MVDR A-LASSO algorithm, along with that of the conventional LASSO and OLS A-LASSO are shown in Figure 6. It is clear from this figure that the MVDR A-LASSO algorithm outperforms both the LASSO and OLS A-LASSO algorithms for all SNR.

**Figure 7:**
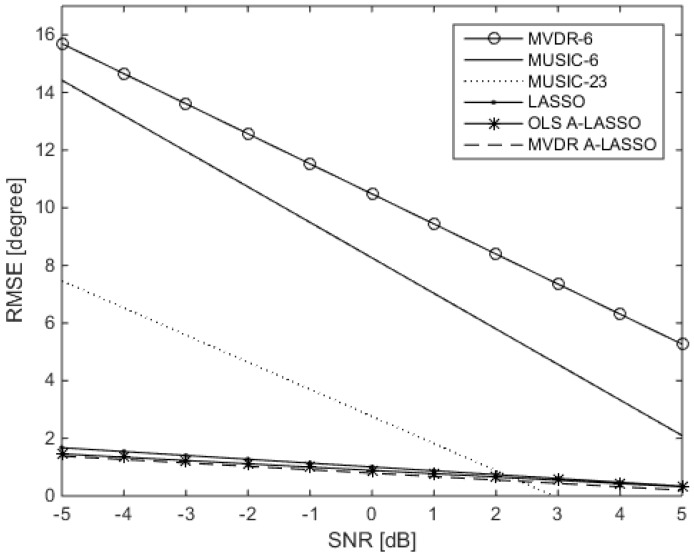
Performance of the LASSO algorithms as SNR is varied in comparison with that of MVDR and MUSIC algorithms, for two source signals at DOAs $60^{\circ }$ and $120^{\circ }$, 10 snapshots and after one iteration of the MVDR A-LASSO and OLS A-LASSO algorithms.

Assuming now that *L* (the number of source signals) is known, we compare the performance of the LASSO algorithms with that of the MVDR algorithm and that of MUSIC. For that purpose, two ULAs, one consisting of six elements and another consisting of 23 elements, are used to evaluate the performance of MUSIC, while only the ULA with six elements is considered for the MVDR algorithm. However, for the three LASSO algorithms, the real array used is as shown in Figure 4, namely with six elements. The performance of the various algorithms as SNR is varied is shown in Figure 7. It is observed from the figure that all three LASSO algorithms outperform the MUSIC algorithm, as well as the MVDR algorithm, even when 23 elements are used in the array.

**Figure 8:**
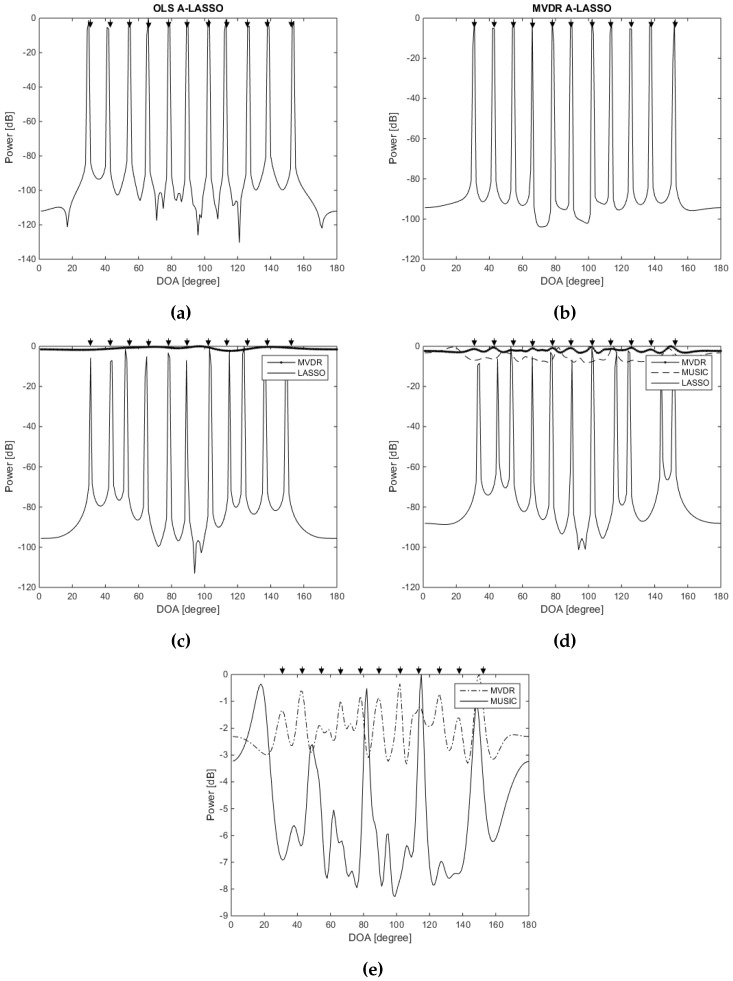
DOA estimation when the number of sources is more than the number of sensors: (**a**) After one iteration of OLS A-LASSO; (**b**) After one iteration of MVDR A-LASSO; (**c**) Classical LASSO and MVDR using a six-element array; (**d**) Classical LASSO, MVDR and MUSIC using a 23-element array; (**e**) MVDR and MUSIC using a 23-element array.

We investigate, in the third simulation, the capabilities of the proposed algorithms in detecting the sources even when the number of sources exceeds the number of physical array elements. In other words, the proposed algorithm is for an underdetermined DOA scenario. For that purpose, two ULAs, one consisting of six elements and another consisting of 23 elements, are used to evaluate the performance of MVDR, while only the ULA with 23 elements is considered for the MUSIC algorithm. However, for the three LASSO algorithms, the real array used is as shown in Figure 4, namely with six elements. Let 11 source signals impinge the array from uniformly-distributed DOAs over $\theta =[30^{\circ },150^{\circ }]$. The snapshots number is chosen to be 70, and SNR is set to be −5 dB. All of the LASSO algorithms can easily identify 11 peaks (even after just one iteration of the MVDR A-LASSO and OLS A-LASSO algorithms), as seen from Figure 8, while MVDR and MUSIC fail to identify the source signals.

**Figure 9:**
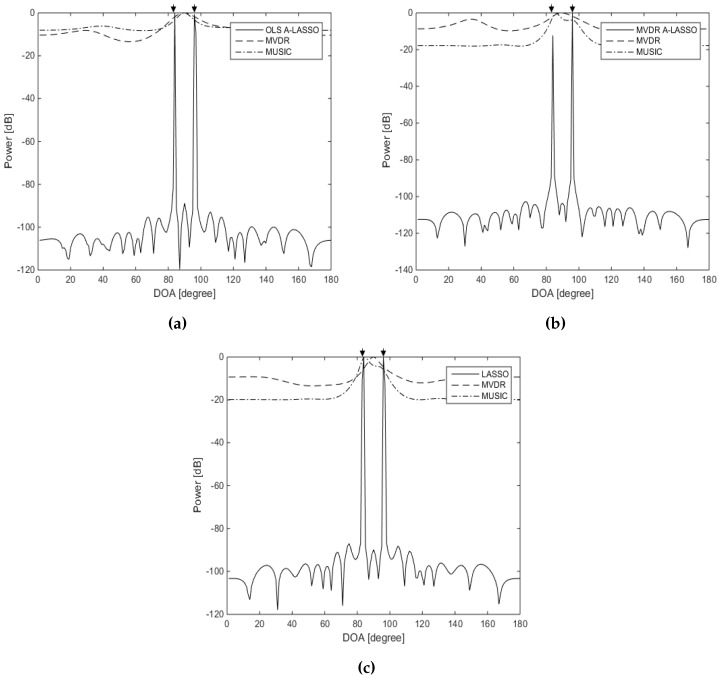
DOA estimation for spatially-closed two-source signals using LASSO algorithms, for two source signals at DOAs $85^{\circ }$ and $95^{\circ }$, 10 snapshots, SNR =15 dB and one iteration of the MVDR A-LASSO and OLS A-LASSO algorithms. (**a**) OLS A-LASSO after the first iteration; (**b**) MVDR A-LASSO after the first iteration; and (**c**) the classical LASSO algorithm.

In the fourth simulation, we examine the resolution of the proposed adaptive algorithms in comparison with that of MVDR and MUSIC. Two spatially-correlated equi-power signals are assumed to impinge on the array from the DOAs of $85^{\circ }$ and $95^{\circ }$. The SNR is set to 15 dB. Figure 9 illustrates the results. Two peaks can easily be identified in the case of the three LASSO algorithms, while in the case of MVDR and MUSIC algorithms, the two peaks are merged into one.

**Figure 10:**
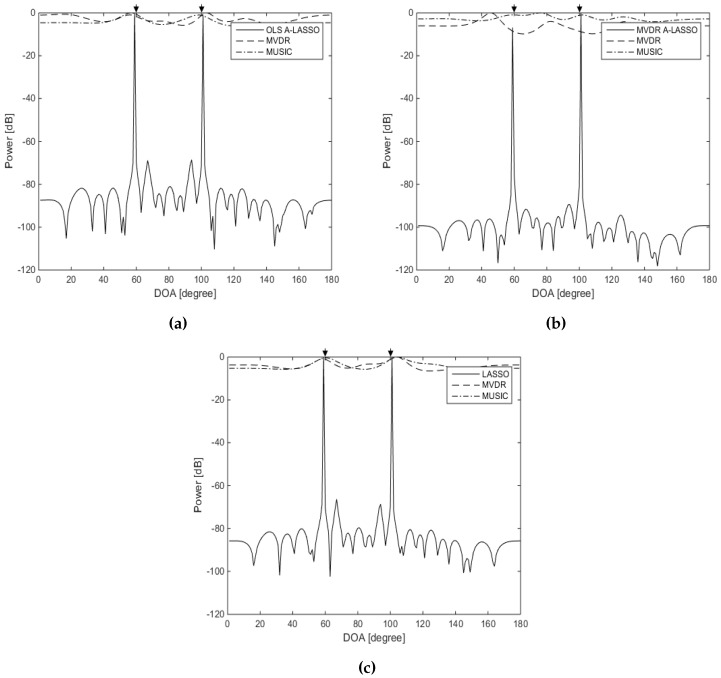
DOA estimation for two correlated source signals using LASSO algorithms, for two source signals at DOAs $60^{\circ }$ and $100^{\circ }$, 10 snapshots, SNR =15 dB and one iteration of the MVDR A-LASSO and OLS A-LASSO algorithms. (**a**) OLS A-LASSO after the first iteration; (**b**) MVDR A-LASSO after the first iteration; and (**c**) the classical LASSO algorithm.

In the fifth simulation, we examine the performance of our proposed algorithms for the detection of correlated source signals. Two fully-correlated (coherent) source signals are assumed to impinge on the array from directions of $60^{\circ }$ and $100^{\circ }$ with SNR set to 15 dB. Figure 10 shows that the three LASSO algorithms can resolve the two sources, revealing the capability of the algorithms in detecting correlated source signals. It is also clear that MVDR and MUSIC fail to distinguish the two sources.

From the above simulations, it is seen that the performance of the A-LASSO-based DOA estimation is superior to that of the MVDR (for which *L* is not required to be known) and that of MUSIC (for which *L* must be known in advance). Further, we also conclude that the performance of the classical LASSO is inferior to that of the two A-LASSO schemes, even though it exhibits a performance better than that of MVDR and MUSIC. In view of these results, we will not consider MVDR, MUSIC or the classical LASSO algorithm further in our study.

#### 5.1.2. Investigations of A-LASSO Algorithms

**Figure 11:**
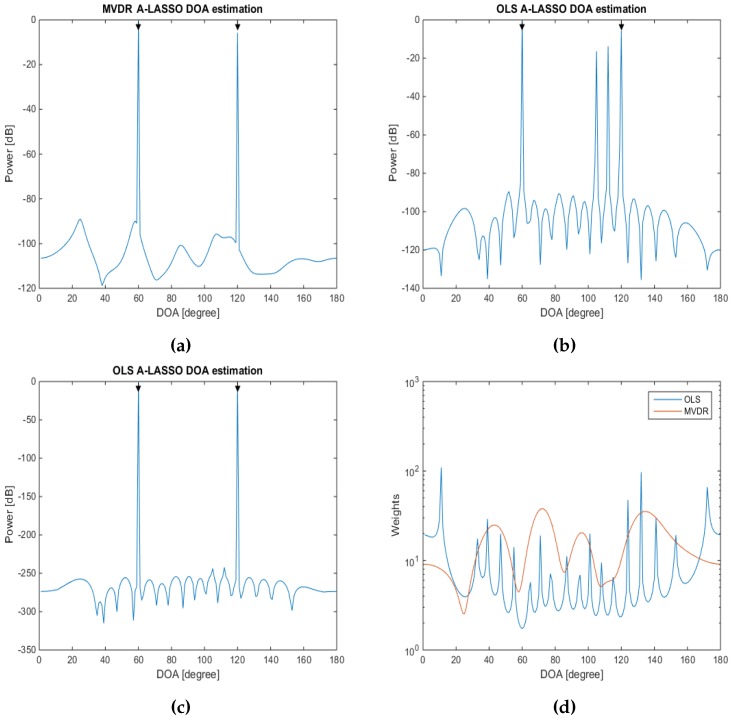
DOA estimation using A-LASSO algorithms, for two source signals at DOAs $60^{\circ }$ and $120^{\circ }$, 10 snapshots, SNR =0 dB. (**a**) MVDR A-LASSO after the first iteration; (**b**) OLS A-LASSO after the first iteration; (**c**) OLS A-LASSO after five iterations; and (**d**) initial weights of the two algorithms.

In the sixth simulation, we test the performance of the OLS A-LASSO and MVDR A-LASSO algorithms in a low SNR situation. For this purpose, we consider two source signals with DOAs of $60^{\circ }$ and $120^{\circ }$, set SNR to 0 dB and snapshots to 10. The results for the two A-LASSO algorithms are shown in Figure 11. It can be seen from this figure that MVDR A-LASSO can detect the source signals after the first iteration itself, while OLS A-LASSO needs more iterations to be able to eliminate all of the false peaks. This can be explained by looking at the initial weights for both the OLS A-LASSO and MVDR A-LASSO algorithms. Figure 11d illustrates the initial weights for OLS A-LASSO and MVDR A-LASSO; it can be seen that the weights using the MVDR A-LASSO algorithm are relatively smooth compared to those of OLS A-LASSO. Furthermore, it can be seen that the OLS A-LASSO weight consists of many peaks that affect its performance and lead to false source signal peaks.

**Figure 12:**
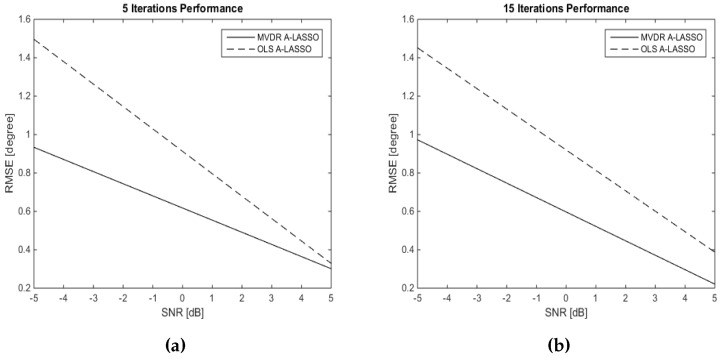
DOA estimation of two source signals at DOAs $60^{\circ }$ and $120^{\circ }$, and 10 snapshots (**a**) after five iterations and (**b**) after 15 iterations, using the A-LASSO algorithms.

The DOA performance is investigated after five and 15 iterations for both the OLS A-LASSO and MVDR A-LASSO in the seventh simulation. Two signal sources are assumed to be impinging the array from DOAs of $60^{\circ }$ and $120^{\circ }$, while the SNR changes. The snapshot number is chosen to be 10; the results are shown in Figure 12. It can be seen from the figure that, in terms of RMSE, MVDR A-LASSO outperforms the OLS A-LASSO algorithms.

Based on the results of the previous simulations (Figure 5, Figure 6, Figure 11 and Figure 12), it is clear that MVDR A-LASSO outperforms OLS A-LASSO. Hence, OLS A-LASSO will not be considered in the rest of the paper.

#### 5.1.3. Investigations of the MVDR A-LASSO Algorithm

**Figure 13:**
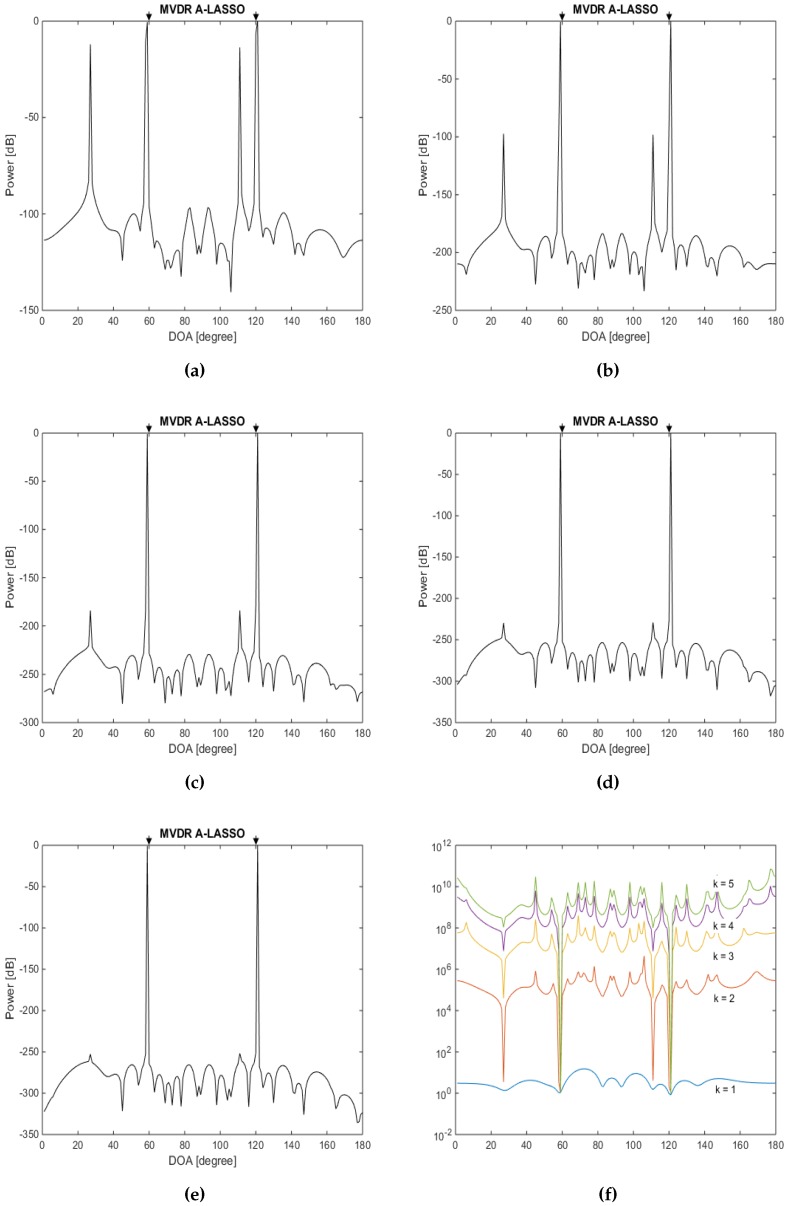
DOA estimation in the case of two source signals at DOAs $60^{\circ }$ and $120^{\circ }$, SNR =−5 dB, 50 snapshots using MVDR A-LASSO algorithm. (**a**–**e**) after 1−5 iterations; and (**f**) MVDR A-LASSO weights as the number of iterations *k* varies.

The eighth simulation investigates the DOA estimation using MVDR A-LASSO as we increase the number of iterations. Let two source signals with DOAs of $60^{\circ }$ and $120^{\circ }$ impinge on the array, and let the SNR be −5 dB and 50 snapshots be used. The results for the first five iterations are shown in Figure 13. It is seen from the figure that, after the first iteration, fake source signal peaks appear. As the algorithm runs, the weights corresponding to the fake source signals become very large, whereas those of the actual source signals remain constant. Hence, the weights corresponding to the false source signals damp the false peaks, while those of the real source signals remain constant. As a consequence, as the number of iterations increases, it is clear that only the real source signal peaks remain. Furthermore, it can be observed that the sidelobe ratio (SLR) after the fifth iteration is more than twice that after the first iteration.

**Figure 14:**
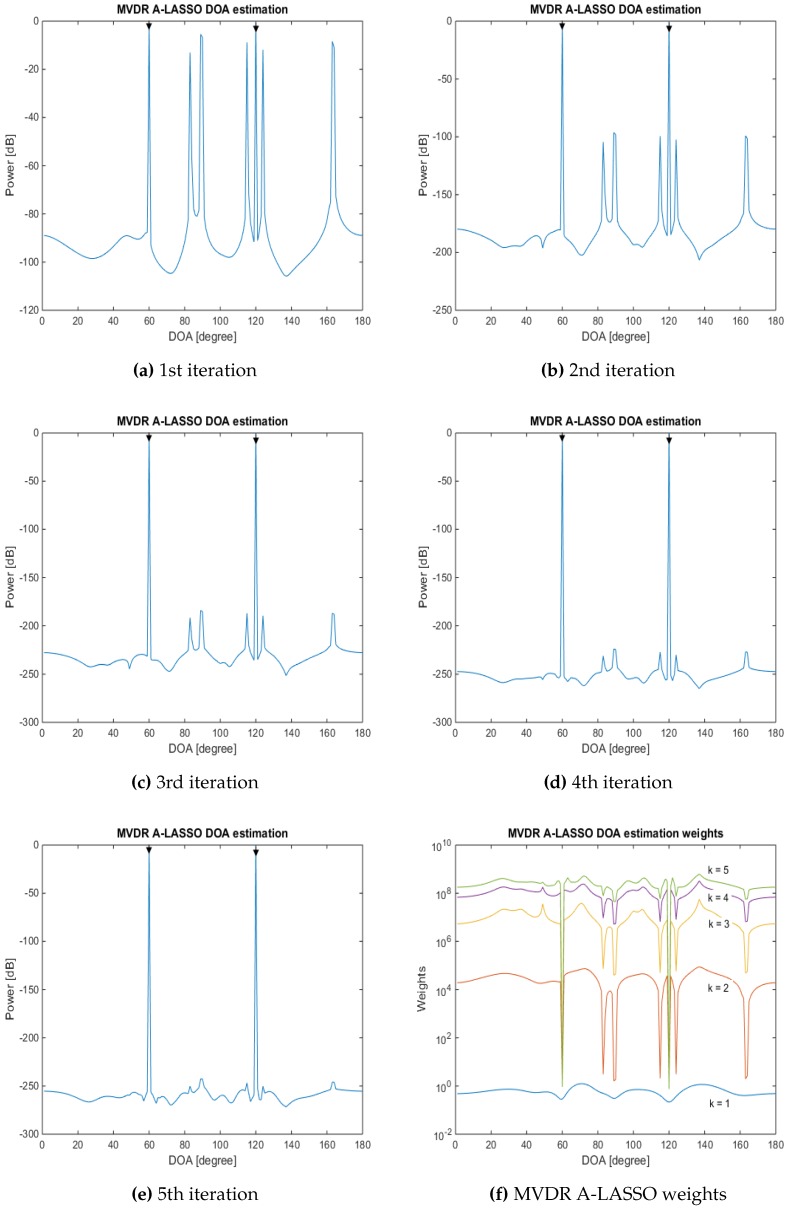
DOA estimation in the case of two source signals at DOAs $60^{\circ }$ and $120^{\circ }$, SNR =−10 dB, 150 snapshots, using the MVDR A-LASSO algorithm. (**a**) to (**e**), after one to five iterations; and (**f**) MVDR A-LASSO weights as the number of iterations *k* varies.

**Figure 15:**
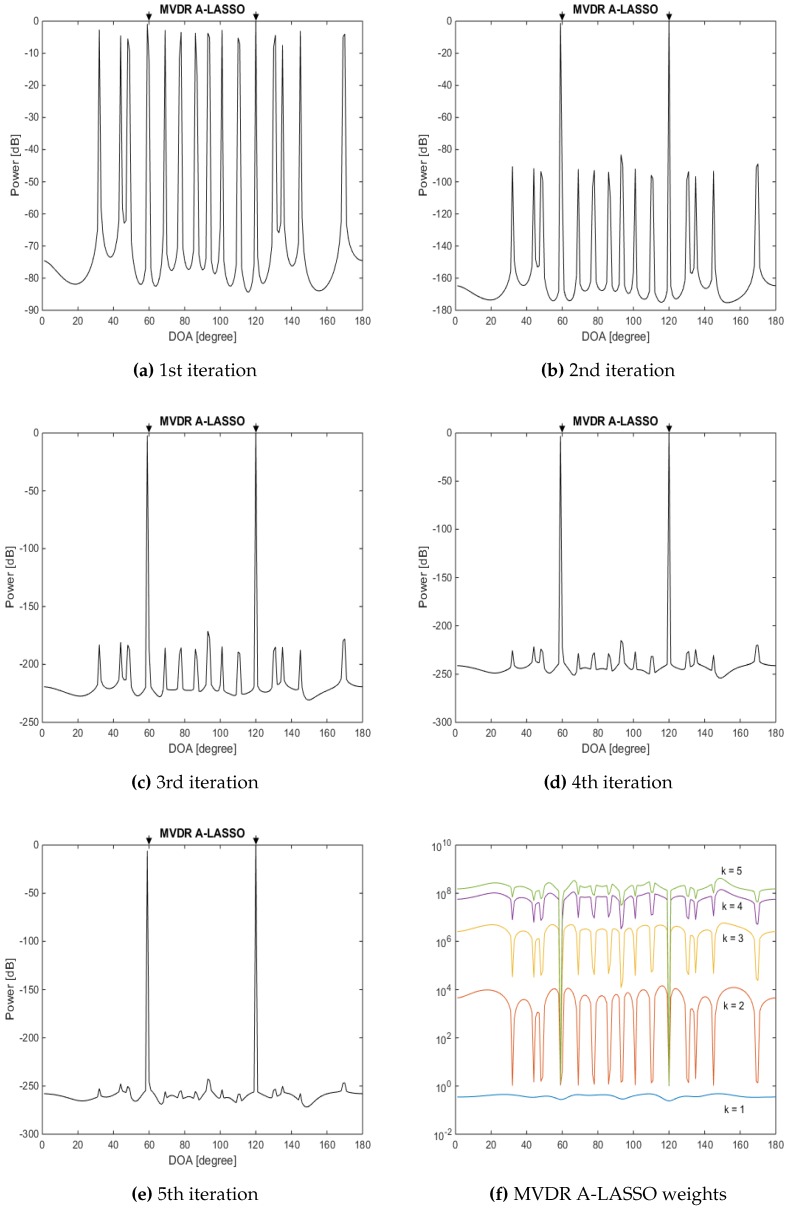
DOA estimation, two source signals at DOAs $60^{\circ }$ and $120^{\circ }$, SNR =−15 dB, 200 snapshots, using the MVDR A-LASSO algorithm. (**a**) to (**e**) after one to five iterations; and (**f**) MVDR A-LASSO weights as the number of iterations *k* varies.

We now examine the performance of the proposed MVDR A-LASSO algorithm at low and very low SNR situations in the ninth simulation. The same settings as in the previous simulation are used, except that the SNR is set to −10 dB and −15 dB. The snapshot number is set to 150 for the first case and 200 for the second one. The results are as shown in Figure 14 for SNR of −10 dB and Figure 15 for that of SNR set to be −15 dB.

It can be seen from Figure 14 and Figure 15 that the two signals can be identified after only five iterations, even for very low SNR conditions. However, more snapshots are needed in this situation. Thus, it is a trade-off between SNR and the number of snapshots required so that the DOA of the source signals can be correctly identified. It is further observed that even after three iterations, we are able to identify the two source signals.

**Figure 16:**
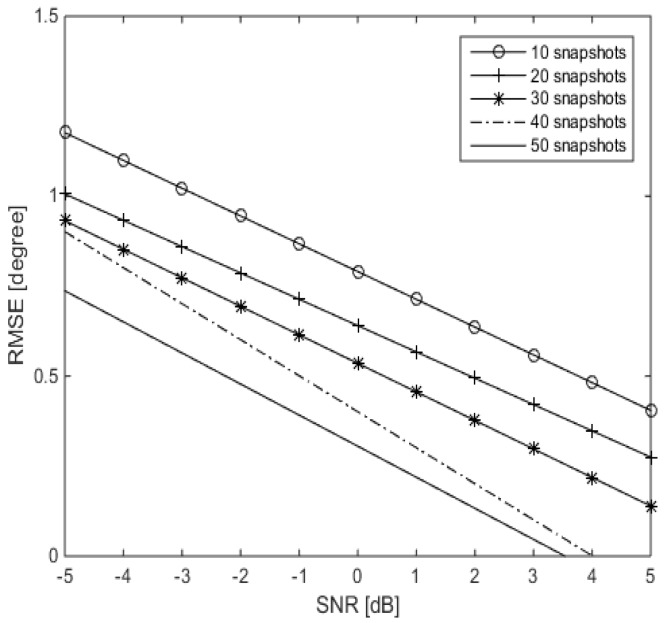
MVDR A-LASSO DOA estimation performance versus the number of snapshots, two source signals with DOAs $60^{\circ }$ and $120^{\circ }$, γ=0.5.

In the tenth simulation, we study the effect of changing the number of snapshots on the performance of MVDR A-LASSO. We consider two source signals arriving from DOAs of $60^{\circ }$ and $120^{\circ }$ with SNR changing from −5 dB to 5 dB. The results are shown in Figure 16. It can be observed from this figure that increasing the number of snapshots leads to an enhancement in the performance, in terms of RMSE. In other words, it is a trade-off between the number of snapshots and the RMSE. For low and very low SNR, the number of snapshots has to be increased, for better performance.

**Figure 17:**
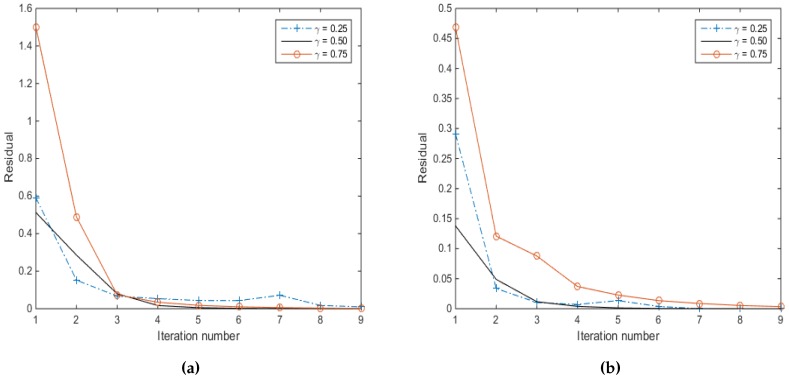
The residual for two source signals at DOAs $60^{\circ }$ and $120^{\circ }$, with γ=0.25,0.5 and 0.75, 10 iterations, SNR =−5 dB using the MVDR A-LASSO algorithm, (**a**) 10 snapshots and (**b**) 50 snapshots.

**Figure 18:**
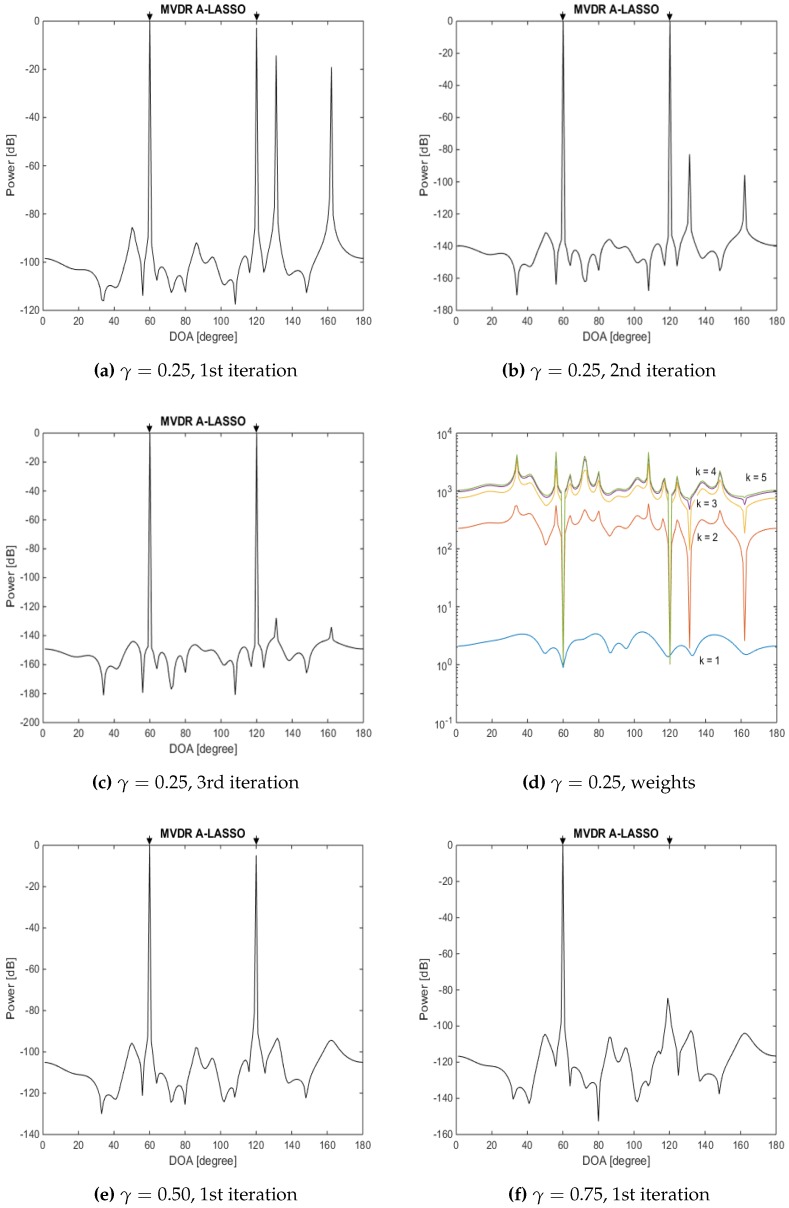
DOA estimation of two source signals at DOAs $60^{\circ }$ and $120^{\circ }$, 50 snapshot, SNR =−5 dB, using the MVDR A-LASSO algorithm, (**a**)–(**d**) γ=0.25; (**e**) γ=0.50; and (**f**) γ=0.75.

The eleventh simulation involves the investigation of the effect of changing *γ*. Two source signals impinging the array from DOAs of $60^{\circ }$ and $120^{\circ }$ are considered, the SNR being set to −5 dB while changing *γ*. Figure 17 shows the residual, *R*, in terms of the absolute value of $\left(\;{∥y-\Phi {\bar{s}}_{k+1}∥}_{2}^{2}-{∥y-\Phi {\bar{s}}_{k}∥}_{2}^{2}\;\right)$, where *k* is the iteration number, for 10 simulations. From this figure, it is clear that increasing the number of snapshots decreases the residuals for all three cases of γ=0.25,0.5 and 0.75. Furthermore, the residual after the end of the first iteration using γ=0.5 is smaller than when γ=0.25 or 0.75, even when the number of snapshots is increased. From Figure 17b, it can be seen that, at the end of the second iteration, the residual corresponding to γ=0.25 is smaller than that corresponding to γ=0.5 or γ=0.75. However, for none of these values of *γ* have we achieved convergence by the end of the second iteration. At the end of Iteration 3, the convergence of the residual is achieved only for the weight factor γ=0.5. However, since the residual for γ=0.5 at the end of the first iteration is less than that of γ=0.25, the signal sources are identified at the end of the first iteration (Figure 18e), while that of γ=0.25 needs more iterations (Figure 18a–c). The effect of *γ* on the DOA estimation, using the same previous source signals, with SNR chosen to be −5 dB is shown in Figure 18. In this case, 50 snapshots are used, while *γ* assumes values of 0.25,0.50 and 0.75. It can be seen from this figure that γ=0.5 provides the right signal sources at the end of the first iteration with a smaller residual than for γ=0.25 or 0.75. On the other hand, selecting γ=0.25 leads to more fake source signals being detected, and as a consequence, more iterations are needed to identify the real sources, while using γ=0.75 leads to misidentifying one of the source signals.

**Figure 19:**
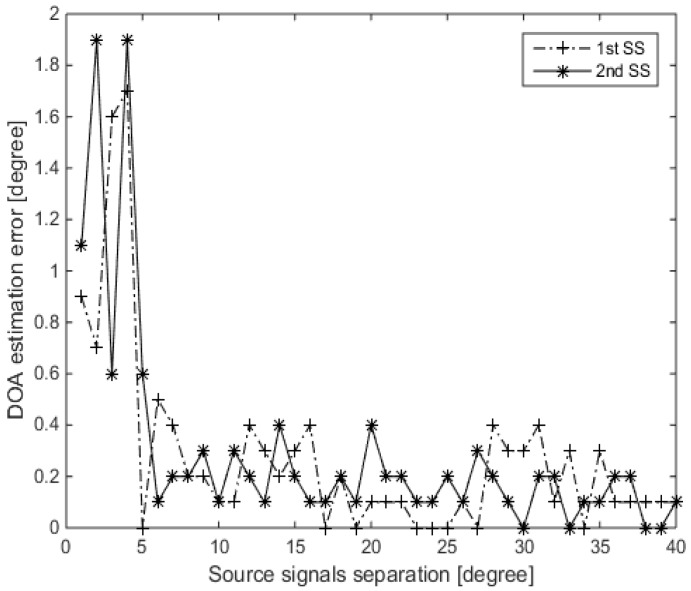
DOA estimation error for two sources as a function of separation between the two sources, SNR = 10 dB, 10 snapshots and one iteration of MVDR A-LASSO.

The twelfth simulation involves the investigation of the effect of varying the angular separation between the source signals. Consider two source signals, the first one held fixed at DOA of $60^{\circ }$, while the second one with DOA ranging from $61^{\circ }$ to $100^{\circ }$ with steps of $1^{\circ }$. The SNR is set to be 10 dB; 10 snapshots are considered for the simulation; 100 trials for each point; and a sampling grid ${\bar{\theta }}_{n}\in \left[1^{\circ }:180^{\circ }\right]$ chosen to be of $0.1^{\circ }$ steps. Figure 19 illustrates the DOA estimation error as a function of the angular separation between the two source signals. It can be seen from this figure that the DOA estimation error is less than $2^{\circ }$ for an angular separation $<5^{\circ }$. This DOA estimation error is reduced to $<0.4^{\circ }$ for an angular separation $\geq 6^{\circ }$.

### 5.2. Wideband Signal Sources

**Figure 20:**
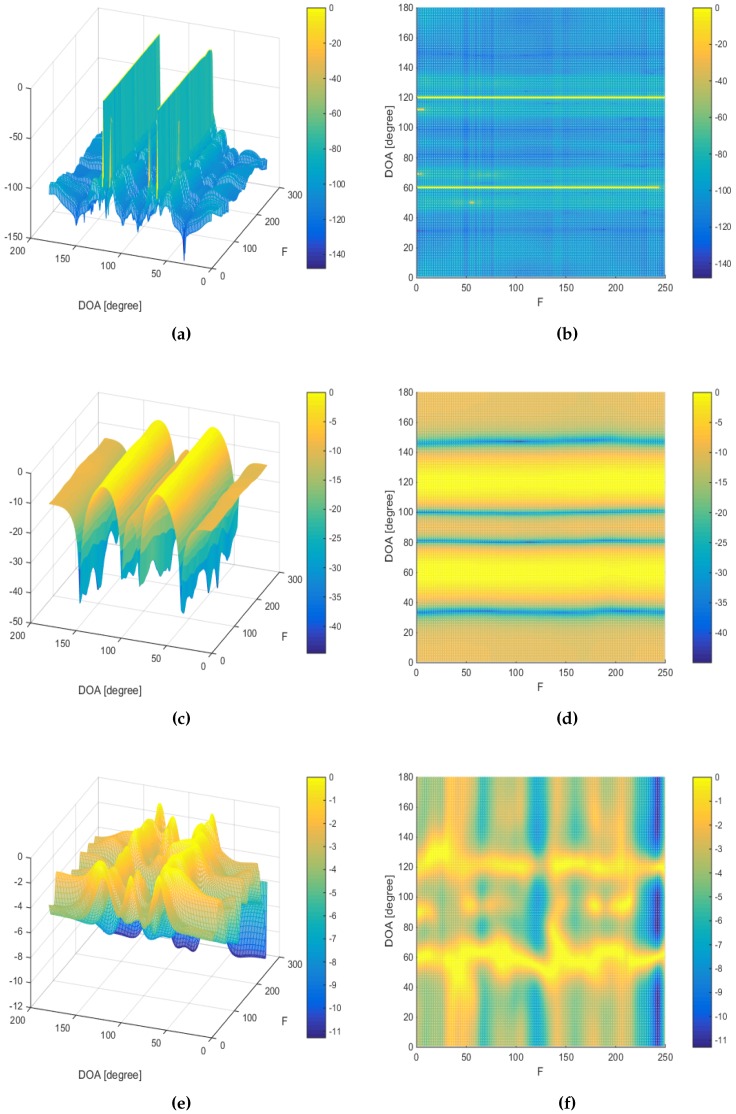
Wideband DOA estimation, two chirp source signals at DOAs $60^{\circ }$ and $120^{\circ }$, γ=0.5, 10 snapshots, using uniform linear array (ULA) containing six sensors for conventional beamforming and the MUSIC algorithm. (**a**,**b**) The MVDR A-LASSO algorithm (after the first iteration); (**c**,**d**) Conventional beamforming; and (**e**,**f**) the MUSIC algorithm.

**Figure 21:**
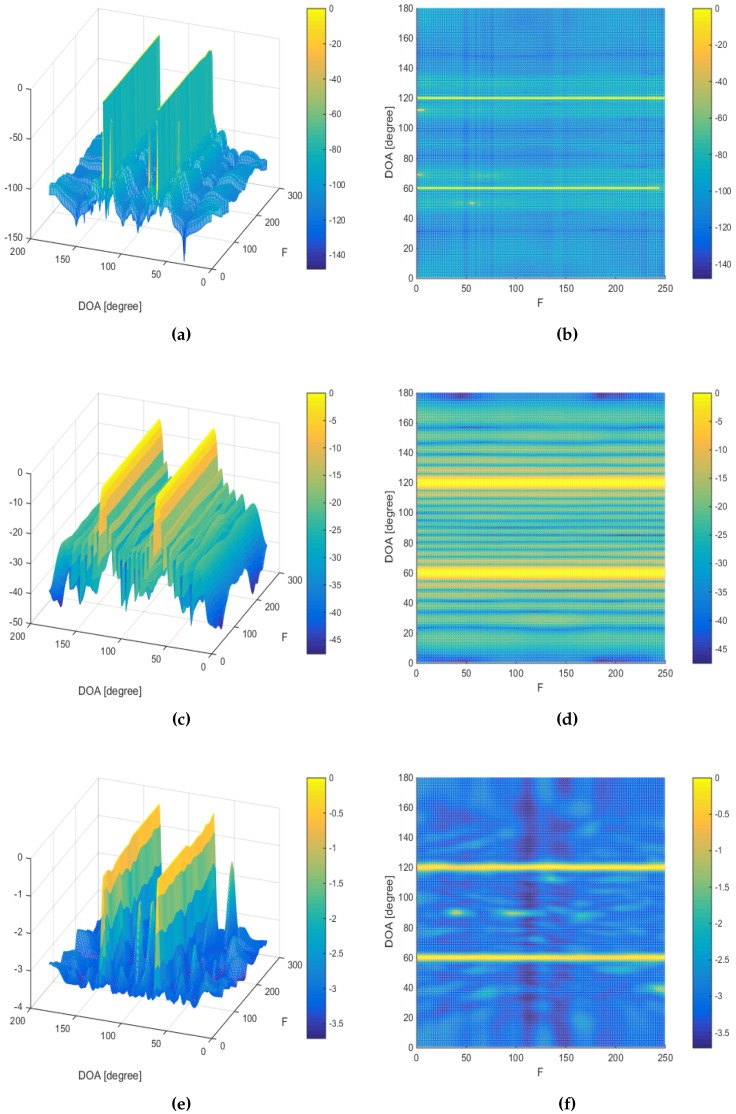
Wideband DOA estimation, two chirp source signals at DOAs $60^{\circ }$ and $120^{\circ }$, γ=0.5, 10 snapshots, using ULA containing 23 sensors for conventional beamforming and MUSIC algorithm. (**a**,**b**) The MVDR A-LASSO algorithm (after the first iteration); (**c**,**d**) Conventional beamforming; and (**e**,**f**) the MUSIC algorithm.

The last simulation is to investigate the DOA estimation in a wideband source signal scenario. Two chirp source signals, with frequency spanning from 250 to 500 Hz, are assumed to be impinging the array from DOAs of $60^{\circ }$ and $120^{\circ }$, with the SNR being set to 30 dB and the number of snapshots being 10. Figure 20 and Figure 21 show the wideband DOA estimation results assuming two ULAs containing six sensors and 23 sensors, respectively. Here, we compare the performance of the proposed MVDR A-LASSO with that of both the conventional beamforming and MUSIC algorithm in a wideband scenario. It can be seen that the conventional beamforming suffers from wide beams so that the source signals can be merged if they are close to each other (Figure 20c,d and Figure 21c,d). Increasing the number of sensors to 23 (the same number of sensors in the virtual array) leads to narrower beams (Figure 21c,d). However, conventional beamforming still has wider beams. Using the MUSIC algorithm, we cannot identify the two source signals using a six-sensor ULA (Figure 20e,f); however, we can identify them using an array with 23 sensors (Figure 21e,f). Using MVDR A-LASSO (Figure 20a,b), we can easily identify the source signals using the sensor array shown in Figure 4. We notice that the sidelobe ratio of the MVDR A-LASSO is superior to that of both the conventional beamforming and MUSIC algorithm. Furthermore, MVDR A-LASSO has a sharper beam compared to that of both the conventional beamforming and MUSIC algorithm. This simulation shows that MVDR A-LASSO is applicable to wideband scenarios, as well as to narrowband situations.

## 6. Conclusions

This paper has presented a novel technique using the compressive sensing framework on a sparse linear array. The ordinary least square adaptable LASSO (OLS A-LASSO) has been applied for the first time for the DOA estimation problem. Further, we have proposed a new adaptable LASSO algorithm, the MVDR A-LASSO, for the DOA estimation problem. The proposed A-LASSO algorithm outperforms the classical LASSO, OLS A-LASSO and the classical DOA estimation techniques. It does not require any a priori knowledge about the number of source signals. The proposed algorithm is able to perform DOA estimation using a smaller number of snapshots and is able to estimate correlated source signals and spatially-related source signals. Moreover, even when the number of source signals is known, it outperforms the MUSIC algorithm. Our proposed algorithm can identify $(\left(M^{2}-2\right)/2+M-1)/2$ source signals using *M* sensors and has a high resolution. Using the proposed technique, we are able to eliminate any spurious peaks and identify only the actual source peaks. Further, it has been shown that, using the proposed MVDR A-LASSO, the source signals can be identified with a lesser number of iterations than that using OLS A-LASSO. Therefore, the computational cost of the MVDR A-LASSO is lower than that of the OLS A-LASSO. Further, the simulations have shown that MVDR A-LASSO is suitable for wideband scenarios, as well. Finally, it is worth mentioning that the proposed scheme for DOA estimation is applicable to other types of array structures, but when the scheme is applied to other structures, it may not provide all of the advantages that it provides to the nested structure.

## Figures and Tables

**Table 1:** Computational complexity of the minimum variance distortionless response (MVDR) algorithm.

Operation	Computation	Cost
Inverse Covariance Matrix	$R_{ss}^{-1}$	$O({\bar{M}}^{3})$
Beamformer Weight	$z=\frac{R_{ss}^{-1}a_{1}\left({\bar{\theta }}_{n}\right)}{a_{1}^{H}\left({\bar{\theta }}_{n}\right)R_{ss}^{-1}a_{1}\left({\bar{\theta }}_{n}\right)}$	$O(2{\bar{M}}^{2}+3\bar{M})$
Beamformer Sum	$w\left({\bar{\theta }}_{n}\right)={\left[a_{1}^{H}\left({\bar{\theta }}_{n}\right)R_{ss}^{-1}a_{1}\left({\bar{\theta }}_{n}\right)\right]}^{-1}$	$O(\bar{M}N)$
